# Fuchs' Uveitis: Failure to Associate Vitritis and Disc Hyperfluorescence with the Disease is the Major Factor for Misdiagnosis and Diagnostic Delay

**DOI:** 10.4103/0974-9233.58424

**Published:** 2009

**Authors:** Nadia Bouchenaki, Carl P Herbort

**Affiliations:** 1 Inflammatory and Retinal Eye Diseases, Center for Ophthalmic Specialized Care (COS); Lausanne, Switzerland; 2 Memorial A. de Rothschild, Clinique Générale-Beaulieu, Geneva, Lausanne, Switzerland; 3 University of Lausanne, Lausanne, Switzerland

**Keywords:** Disc Hyperfluorescence, Diagnostic Delay, Fuchs' Uveitis, Fundus Fluorescein Angiography, Laser Flare Photometry, Vitritis

## Abstract

**Purpose:**

Fuchs' uveitis is often diagnosed with substantial delay at the origin of deleterious consequences such as unnecessary treatment. The aim of the study was to analyse the type and frequency of posterior inflammatory and fluorescein angiographic signs in Fuchs' uveitis in conjunction with the other clinical signs and evaluate their respective importance in the diagnosis of the disease. In particular, diagnostic delay and erroneous diagnoses were investigated.

**Patients and Methods:**

Patients seen in our centers between 1995 and 2008 with the diagnosis of Fuchs' uveitis were analysed. The data collected included age, initial and final visual acuities, clinical findings at presentation, mean diagnostic delay, erroneous diagnoses, laser flare photometry values, fundus and fluorescein angiography manifestations and ocular complications.

**Results:**

One hundred and five patients were included. The mean age at diagnosis was 34 years. Twelve patients (11.4%) had bilateral involvement. The mean diagnostic delay was 3.04 ± 4.30 years. The most frequent clinical signs were vitreous infiltration (97.40%), typical Fuchs' keratic precipitates (94.90%), crystalline lens opacities or cataract (47%), heterochromia (42.60%), ocular hypertension or glaucoma (12.80%). The mean laser flare photometry value at presentation was 9.85 ± 6.28 ph/ms. Thirty-nine patients (37.14%) had undergone fluorescein angiography showing disc hyperfluorescence in 97.7% and peripheral retinal vascular leakage in 13.6%.

**Conclusions:**

Fuchs' uveitis is significantly underdiagnosed likely because vitreous involvement was previously described but not commonly recognized as an association with Fuchs' uveitis in the clinician's mind and therefore has often been given a different diagnostic label. Moreover, the very frequent inflammatory signs on fluorescein angiography such as disc hyperfluorescence and more rarely peripheral retinal vascular leakage, which has not been typically associated with Fuchs' uveitis, appear to represent an additional factor leading to misdiagnosis. Such clinical findings need to be publicised in order to reduce misdiagnosis, and diagnostic delay.

## INTRODUCTION

Since the first description of Fuchs' uveitis by Ernst Fuchs a century ago,^1^,^2^ numerous reports have been written about this disease globally including Europe,^3^–^11^ the Middle East 12,13 Asia^14^,^15^ and North and South Americas.^16^–^19^ Despite the fact that Fuchs originally described the main clinical signs to include fine retrodescemetic keratic precipitates (KPs), vitreous infiltration, absence of iris inflammation, atrophic iris changes, lens opacification and good tolerance to surgery^2^, Fuchs' uveitis often remains underdiagnosed and/or misdiagnosed leading to unnecessary investigations and therapy.^7^,^10^,^11^,^17^,^20^,^21^ The aetiology of Fuchs' uveitis remains unknown; however, recent studies have reported an association of Fuchs' uveitis with the rubella virus.^22^,^23^ as well as with ocular herpes.^24^,^25^ The diagnosis of Fuchs' uveitis is based on clinical examination with the presence (or absence) of clinical signs, which may be subtle, or some signs absent at initial presentation; or may be missed or misinterpreted leading to misdiagnosis and diagnostic delay. This is especially the case for posterior segment manifestations. For example, vitreous infiltration, disc staining and retinal capillary leakage on fundus fluorescein angiography are generally not associated with Fuchs' uveitis, leading the clinician towards an incorrect diagnosis. 

The aim of the present study was to determine the characteristics of Fuchs' uveitis from our patient base with specific attention directed at investigating the causes of Fuchs' uveitis being misdiagnosed, the causes of diagnostic delay and the type of erroneous diagnoses.

## MATERIALS AND METHODS

We retrospectively reviewed the medical records of all patients with the diagnosis of Fuchs' uveitis seen at the Center for Ophthalmic Specialized Care (COS) in Lausanne and at Memorial A. de Rothschild, Clinique Générale-Beaulieu in Geneva, Switzerland between 1995 and 2008. A complete ocular examination was performed including Snellen visual acuity, slit-lamp examination, applanation tonometry, dilated funduscopy, laser flare photometry and gonioscopy. Some patients had fundus fluorescein angiography already performed elsewhere prior to presentation at our centers. In some cases angiography was performed to confirm absence of cystoid macular edema as an additional element of Fuchs' uveitis.

Data on the onset of disease, presenting symptoms, the nature of uveitis, the wrong diagnoses and treatments received were compiled. Detailed clinical information regarding the signs found in Fuchs' uveitis such as laterality, the presence and disposition of KPs, heterochromia, iris structure, presence of Koeppe nodules, the presence of abnormal vessels in the irido-corneal angle, the presence of opacities in the crystalline lens, vitreous infiltration, fundus lesions and/or scars and anterior and posterior segment complications were recorded.

Laser flare photometry (Kowa, Japan) was performed in all our patients at presentation and during follow-up. This technology gives objective and precise information on the level of anterior chamber (intraocular) inflammation and is especially useful when recording low-grade subclinical intraocular inflammation.

## RESULTS

### General findings and epidemiologic data

Between 1995 and 2008, 105 of 1240 new patients (8.46%) seen in the uveitis clinic at the Center for Ophthalmic Specialized Care (COS) in Lausanne and at the Memorial A. de Rothschild, Clinique Générale-Beaulieu in Geneva were diagnosed with Fuchs' uveitis. The proportion of referred patients was 92.4%. There were 52 males and 53 female patients, and the mean age at diagnosis was 34 years (range from 6 to 75 years). In 12 of 105 patients (11.40%), involvement was bilateral giving a total of 117 Fuchs eyes included in the study. In 75 of 105 patients (71.40%), diagnosis was not reached within one month after presentation to the ophthalmologist and the mean duration of the diagnostic delay was 3.04 ± 4.3 years (range, 1 month to 24 years).

In 77.10% of patients Fuchs' uveitis was mistaken for a uveitis of the posterior segment. The most frequent erroneous diagnoses were intermediate uveitis in 56.80% of cases, posterior uveitis in 8.10% and panuveitis in 12.20%. In 22.30%, the erroneous diagnosis was other forms of anterior uveitis including herpetic uveitis, granulomatous anterior uveitis or Posner-Schlossmann's syndrome. Treatments prescribed prior to the diagnosis of Fuchs' uveitis included: Systemic therapy in 29 patients (38.70%), mainly oral steroids in 25 patients (86.20%) with additional immunosuppressive therapy in 5 patients (17.20%), systemic antibiotic therapy (against toxoplasmosis or Bartonella) in 3 patients (10.30%) and 3 patients (10.30%) were treated with antiviral agents; periocular steroid injections were used in 8 patients (10.7%) of the non-diagnosed patients; 44 patients (41.9%) received topical corticosteroids.

### Clinical symptoms and signs

The most common ocular symptoms included floaters, a decrease in visual function or eye discomfort. The majority of patients that presented sought ophthalmological care because symptoms prompted them to request a consult. The leading symptoms were floaters in 49.50% of patients, followed by reduced visual function in 35.20% and eye discomfort or pain in 3.80%. In 7.60% of the patients the ocular pathology was discovered during a routine eye examination. 

Mean initial visual acuity was 0.86 (range, 0.05 to 1.50). Visual acuity was higher than 0.50 in 95 eyes, with a majority (85 eyes) presenting a good acuity of over 0.7. Twenty two eyes presented with a visual acuity inferior or equal to 0.5, due to the presence of a cataract (11 eyes), secondary cataracts (2 eyes), dense vitreous haze (2 eyes), post-surgical cystoid macular edema (1 eye), severe glaucoma (2 eyes), toxoplasmosis scars (2 eyes) and amblyopia (2 eyes). Mean final visual acuity was 0.96 (range 0.2 to 1.5).

**Figure 1:**
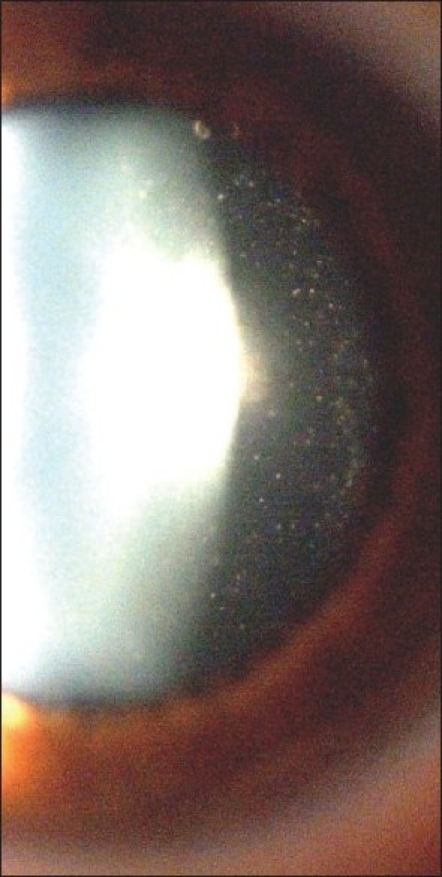
Posterior polar crystalline opacity and vitreous infiltration. Vitreous infiltration is seen to the right of posterior aspect of the slit

The most frequent clinical sign found was vitreous infiltration present in 114 of 117 eyes (97.4%) [Figure 1]. The degree of vitreous involvement ranged from slight with a few cells (46.15%) to moderate (34.19%) and severe (14.53%) with dense vitreous strands and condensations. 

**Figure 2:**
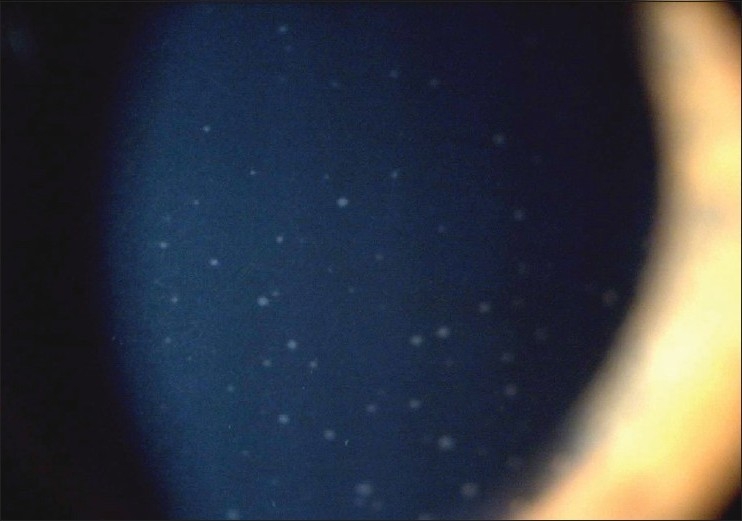
Typical Fuchs' keratic precipitates distributed over the cornea

The characteristic stellate keratic precipitates (KPs) or microgranulomatous KPs were present in 94.9% of eyes (111/117 eyes) and their distribution was not limited to the inferior cornea [Figure 2]. Keratic precipitates could be absent at presentation, appearing during follow-up and could also disappear during follow-up.

Opacities of the crystalline lens, mostly posterior subcapsular opacification were recorded at presentation in 47% of eyes (55 eyes) [Figure 1]. Twelve percent of eyes (14 eyes) had undergone cataract surgery prior to referral and prior to the diagnosis of Fuchs' uveitis. 

Heterochromia and/or major iris texture changes were present in 42.6% of the patients. This low proportion is explained by the fact that brown irides do not often become hypochromic.

**Figure 3:**
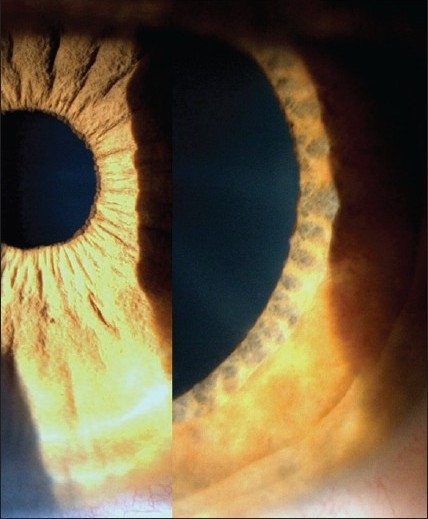
Texture changes in brown iris. Normal structure in the right eye shown in the left photograph. The left eye (right picture) shows loss of pigment at the pupillary margin and loss of crypts on the iris surface

This sign has been overrated in the literature as it is a prominent finding in blue-colored and bi-colored irides, which is characteristic of the Viennese population where the disease was first described. Brown irides should be examined for texture changes, which are present in a high proportion of cases [Figure 3]. They were likely overlooked in this retrospective study.

Koeppe nodules were seen in 13.3%. Gonioscopy was performed in 25 patients showing abnormal vessels in the irido-corneal angle in 13 eyes (52%). None of the eyes examined showed posterior synechiae. The consistency of this finding is a strong diagnostic criterion for Fuchs' uveitis.

The mean intraocular pressure of eyes with Fuchs' uveitis at presentation was 12.95 ± 4.49 mm Hg, whereas the mean pressure of the healthy fellow eye was 13.54 ± 2.20 mmHg. The intraocular pressure of the affected eye was lower than the healthy fellow eye in 50% of the patients, equal in both eyes in 31.3% and higher than the normal eye in 18.8%. However, 55.5% of the latter cases, presented with glaucoma or ocular hypertension in the affected eye.

Fundus examination was unremarkable in 69 eyes (59%) and difficult to visualize in 15 eyes (12.8%) due to the presence of a vitreous haze or cataract. Papillary hyperemia or blurred disc margins were present in 12 eyes (10.3%), pale atrophic discs were present in 2 eyes (1.7%). A chorioretinal scar was present in 7 eyes (6.0%) of which 4 eyes had peripheral scars, 2 eyes had macular scars and in one eye it was located in the peripapillary region. Seven patients (6.0%) developed a peripheral hole or tear during follow-up. Epiretinal fibrosis was detected in 3 eyes (2.6%). One fundus (0.9%) showed a peripheral nevus and other an inferior peripheral schisis (0.9%). Cystoid macular edema seen funduscopically was present in one patient who had undergone cataract surgery.

### Laser flare photometry results

Laser flare photometry measurements were available in 103 eyes. The breakdown of the blood aqueous barrier, measured by laser flare photometry was minimal, amounting to 9.85 ± 6.28 ph/ ms at presentation showing a slight disruption of the blood-aqueous barrier and remaining stable at 9.98 ± 6.02 ph/ms at the end of follow-up period.

### Fundus fluorescein angiographic findings

**Figure 4:**
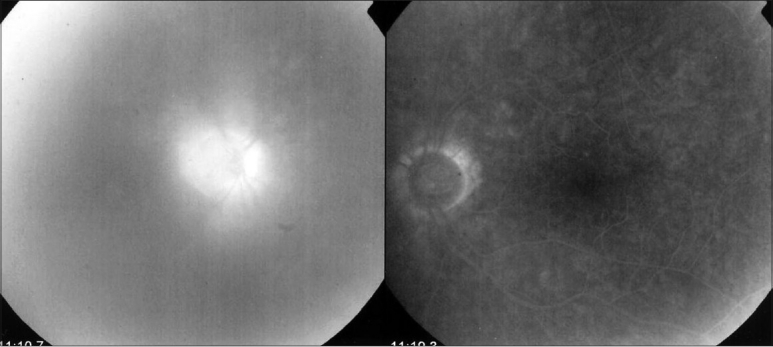
Disc hyperfluorescence. Disc staining in the affected right eye shown in the left side compared to the normal fellow eye. Also note the vitreous infiltration

Fundus fluorescein angiography was performed in 39 patients (37.14%) mainly prior to the correct diagnosis. Taking into account five bilateral cases, a total of 44 eyes had a fundus fluorescein angiography that were suitable for this study. Disc hyperfluorescence was present in all but one patient (97.7%) [Figure 4]. The degree of disc hyperfluorescence was not correlated with the degree of vitreous infiltration and did not change appreciably on successive angiographies and was symmetrical in bilateral cases. Mild mid-peripheral retinal vascular leakage was present in 13.6%. Cystoid macular edema occurred in 15.2% of cases all occurring after cataract surgery. Eyes that had not undergone surgery did not present with CME. Hence, the absence of angiographic macular edema in longstanding uveitis with vitreous infiltration was considered confirmatory for a diagnosis of Fuchs' uveitis. 

### Complications in the anterior and posterior segments

A total of 33 eyes (28.2%) underwent cataract surgery. Fourteen eyes underwent cataract surgery before referral. Two of these eyes did not undergo intraocular lens implantation. Nineteen eyes underwent phacoemulsification with lens implantation after the diagnosis of Fuchs' uveitis, during follow-up. Cystoid macular edema occurred in five patients' post-cataract surgery (15.2%) and was detected by fluorescein angiography in all cases except in one case where it was obvious during funduscopy.

Fifteen eyes (12.8%) presented with either ocular hypertension or glaucoma. Twelve eyes were controlled with topical glaucoma medications and three eyes required surgery. Ocular hypotony was present in one aphakic patient, secondary to partial ciliary body detachment detected by ultrasound biomicroscopy.

Seven patients (6%) developed a peripheral hole or tear that had to be treated by laser photocoagulation. Two other patients presented with a retinal detachment and underwent vitrectomy and scleral buckling. Vitrectomy was also performed to clear the vitreous for visually disturbing floaters in two eyes and used as a diagnostic procedure in one patient.

## DISCUSSION

Our study shows that the diagnosis of Fuchs' uveitis can be delayed in up to 70% of cases during a mean of more than three years. Previous studies have shown similar^10^,^11^,^15^ or even greater (6.3 years) delays of diagnosis.^7^ Uveitis of the posterior segment represented the majority of erroneous diagnoses in a proportion of over 70% of our non-diagnosed cases. In almost 50% of our patients the main symptom at presentation was the perception of floaters, correlated with the high frequency of vitreous infiltration, which was the most consistent clinical finding present in 97.4%. One drawback of this study is the potential for selection bias due to the high rate of patient referral (more than 90%). This high referral rate due the perception by the general ophthalmologists that vitreous involvement was not associated with Fuchs' uveitis therefore leading to other diagnoses. In most publications, Fuchs' uveitis is identified as an anterior uveitis and it is the anterior involvement that is described and analysed in detail, with only minor regard for vitreous involvement although it is cited in many papers.^5^,^7^,^9^,^14^,^16^,^17^ Lack of consideration of vitreous infiltration as a major diagnostic criteria for Fuchs' uveitis comes from the fact that after Fuchs described vitreous involvement as being a major sign in his original description,^2^ attention was mainly directed to the remarkable anterior signs neglecting vitreous pathology. In their recent series Norsell *et al*.^10^ came to similar conclusions, pointing out the high frequency of vitreous signs, present in more than 90% and implicating this as the origin of most diagnostic and therapeutic problems. Other vitreous features that are usually understated are the peripheral retinal breaks and retinal detachments occurring as a consequence of the vitreous inflammation that was present in 8.5% of our patients.

Stellate KPs were the second most frequent sign after vitreous infiltration, being present in almost all of our patients (95%). This finding is similar to previous studies which also indicate stellate KPs as a consistent finding. However, it should be recognized that the presence of stellate KPs may vary including being absent in the early phase of the disease and may sometimes disappear during follow-up.

Furthermore we demonstrated that heterochromia, a feature strongly associated with Fuchs' uveitis, was present in less than half of the cases. The lower frequency of this sign in our study is likely related to the higher proportion of brown irises present in a fair proportion of patients referred from southern Europe. Other series have reported the low frequency of heterochromia in their dark eyed populations.^11^,^14^,^15^,^17^–^19^ This indicates heterochromia is far less important than vitreous involvement. Overemphasis of heterochromia as a sign in Fuchs' uveitis is explained by the fact that the disease was first described in a geographic area where blue or bi-colored eyes, prone to developing heterochromia, were frequent, so that the term was even included in the naming of the disease-‘Fuchs’ heterochromic cyclitis'. Currently conventional thought is that individuals with brown irides diagnosed with Fuchs' uveitis do not develop heterochromia but changes in iris texture do occur. Therefore we believe it is important to rename this disease “Fuchs' uveitis” as heterochromia is not a significant association.

Subcapsular crystalline lens opacification or cataract described in Fuchs' uveitis was present in nearly half of our patients. It may appear early in the evolution of the disease and the presence of a unilateral cataract in young patients should alert the ophthalmologist to the possibility of a Fuchs' uveitis and spur the search for other clinical signs of this diagnosis. Cataract surgery in Fuchs' uveitis patients does not seem to be more complex than in normal eyes, except that bleeding from angle vessels can occur (Amsler's sign). Ernst Fuchs described good tolerance of eyes with Fuchs uveitis to surgery and excellent visual outcome postoperatively.^2^ However, the possibility of CME postoperatively clearly exists as seen in our study, and it is possible that these eyes are a greater risk of developing CME postoperatively. For example, 5 of 33 operated eyes (15.1%) had CME versus no occurrence in eyes that did not undergo surgery. The absence of angiographic CME in longstanding uveitis with pronounced vitreous infiltration in eyes that did not undergo surgery should be considered as confirmatory criteria of the diagnosis of Fuchs' uveitis.

The mean intraocular pressure was within normal limits in the majority of cases. Also the mean intraocular pressure was lower in most affected eyes when compared with the healthy eye despite a rate of 12.8% of hypertension or glaucoma. Similar to recent Swedish^10^ and Turkish^11^ studies, the rate of ocular hypertension or glaucoma was rather low and rarely needing filtering surgery when compared to other series that reported that filtering surgery was noted in more than 30% of cases.^16^,^17^,^19^

The laser flare photometry results (9.84 ph/ms at presentation) and their stability during follow-up corroborate with previous results^10^ that showed a relatively mild and stable breakdown of the blood-aqueous barrier even after cataract surgery.^11^,^26^–^28^

Two recent studies^11^,^15^ mentioned the presence of disc staining and mid-peripheral retinal leakage in Fuchs' uveitis. We have previously published^29^ the results of fundus fluorescein angiography manifestations in the 39 patients (37.14%). We described disc hyperfluorescence and less frequently, peripheral vascular retinal leakage; and CME present in a moderate proportion of cases after cataract surgery. The high frequency of disc hyperfluorescence (97.1%) on fundus fluorescein angiography could be due to an inflammatory breakdown of the blood-ocular barrier, or secondary to mechanical traction by a heavily infiltrated vitreous proximal to the optic disc. However, optic disc hyperfluorescence was not proportional to vitreous infiltration and pronounced disc hyperfluorescence can be found even in a very mildly infiltrated vitreous.

It is of paramount importance to diagnose Fuchs' uveitis as early as possible in order to avoid unnecessary work-up, and more importantly to avoid ineffective therapy. In most instances the condition is benign and does not require any anti-inflammatory therapy.^20^,^21^ As shown in our study, more than a third of the non-diagnosed patients were exposed to systemic corticosteroids and in some cases, immunosuppressive therapy with the concomitant risk of significant side effects. Furthermore, once the diagnosis is known, the patients can be reassured and yet remain motivated to adhere to regular follow-up visits in order to monitor lens opacities and intraocular pressure as these patients are prone to developing ocular hypertension or glaucoma.^30^

In summary, in addition to the usual known features of Fuchs' uveitis, such as characteristic KPs, heterochromia, lens changes, Koeppe nodules, other less recognized but frequent signs can be helpful to make the diagnosis and should be added to the usual diagnostic criteria. These include vitreous infiltration, absence of posterior synechiae, absence of CME despite prolonged inflammatory involvement, iris texture changes all of which were present in close to 100% of our cases. Clinically, the most important conclusion of this study is the fact that vitreous infiltration is indeed part of the classic picture of Fuchs' uveitis, being the most frequent sign and recognition is imperative in order to avoid unnecessary, possibly deleterious therapy by ophthalmologists unaware of this fact.
